# Developing and user-testing Decision boxes to facilitate shared decision making in primary care - a study protocol

**DOI:** 10.1186/1472-6947-11-17

**Published:** 2011-03-09

**Authors:** Anik Giguere, France Legare, Roland Grad, Pierre Pluye, François Rousseau, R Brian Haynes, Michel Cauchon, Michel Labrecque

**Affiliations:** 1 Research Center of the CHUQ, Saint-Francois d'Assise Hospital, Quebec City, Canada; 2 Dept de médecine familiale et de médecine d'urgence, University Laval, Quebec city, Canada; 3 Dept of Family Medicine, McGill University, Montreal, Canada; 4 Dept of Clinical Epidemiology and Biostatistics and Department of Medicine, McMaster University, Hamilton, Canada

## Abstract

**Background:**

Applying evidence is one of the most challenging steps of evidence-based clinical practice. Healthcare professionals have difficulty interpreting evidence and translating it to patients. Decision boxes are summaries of the most important benefits and harms of diagnostic, therapeutic, and preventive health interventions provided to healthcare professionals before they meet the patient. Our hypothesis is that Decision boxes will prepare clinicians to help patients make informed value-based decisions. By acting as primers, the boxes will enhance the application of evidence-based practices and increase shared decision making during the clinical encounter. The objectives of this study are to provide a framework for developing Decision boxes and testing their value to users.

**Methods/Design:**

We will begin by developing Decision box prototypes for 10 clinical conditions or topics based on a review of the research on risk communication. We will present two prototypes to purposeful samples of 16 family physicians distributed in two focus groups, and 32 patients distributed in four focus groups. We will use the User Experience Model framework to explore users' perceptions of the content and format of each prototype. All discussions will be transcribed, and two researchers will independently perform a hybrid deductive/inductive thematic qualitative analysis of the data. The coding scheme will be developed a priori from the User Experience Model's seven themes (valuable, usable, credible, useful, desirable, accessible and findable), and will include new themes suggested by the data (inductive analysis). Key findings will be triangulated using additional publications on the design of tools to improve risk communication. All 10 Decision boxes will be modified in light of our findings.

**Discussion:**

This study will produce a robust framework for developing and testing Decision boxes that will serve healthcare professionals and patients alike. It is the first step in the development and implementation of a new tool that should facilitate decision making in clinical practice.

## Background

Evidence-based medicine refers to the conscientious, explicit, and judicious use of current best evidence when making decisions about health care [1]. Practicing evidence-based medicine means integrating a practitioner's personal clinical expertise with the best clinical evidence available from scientific research [1]. Applying evidence in clinical practice is one of the most challenging aspects of evidence-based practice, in part because physicians find it difficult to interpret evidence [2]. More specifically, physicians do not always correctly estimate the benefits and harms of the interventions they commonly recommend [3]. Physicians are also unclear about how best to discuss the benefits and harms of treatment with patients [4], this despite evidence that how health professionals present clinical information influences patients' perception of risks-and ultimately, decisions about treatment [5,6].

It is clear, then, that strategies to help physicians easily access and understand evidence of the benefits and harms of healthcare interventions are needed if physicians are to better communicate this knowledge to their patients. Shared decision making (SDM) is a promising avenue for applying scientific evidence in clinical practice. SDM help healthcare professionals and patients make joint decisions based on two elements: the best evidence of the benefits and harms of all available options, and patients' values and preferences in regard to those options, benefits, and harms [7]. By clarifying values and improving patients' feeling of being adequately informed [8], SDM reduces the overuse of screening or treatment options not clearly associated with health benefits for all [9]. Patients' active participation in decision making has also been associated with favourable outcomes such as better quality of life [10] and higher patient satisfaction [11-13] without increasing consultation times [14].

SDM can be facilitated using patient decision aids. Patient decision aids are "interventions designed to help patients make specific and deliberative choices among options (including the status quo) by providing (at the minimum) information on the options and outcomes relevant to a person's health status and implicit methods to clarify values," in particular values about the benefits and harms of the options [8]. Most patient decision aids are designed so that patients can work through them on their own (at home or somewhere else outside of the consultation): for that reason, they can generally be described as patient-mediated interventions. Only a few have been designed for the clinician to use to facilitate decision making at the point of care [15-17].

**Figure 1:**
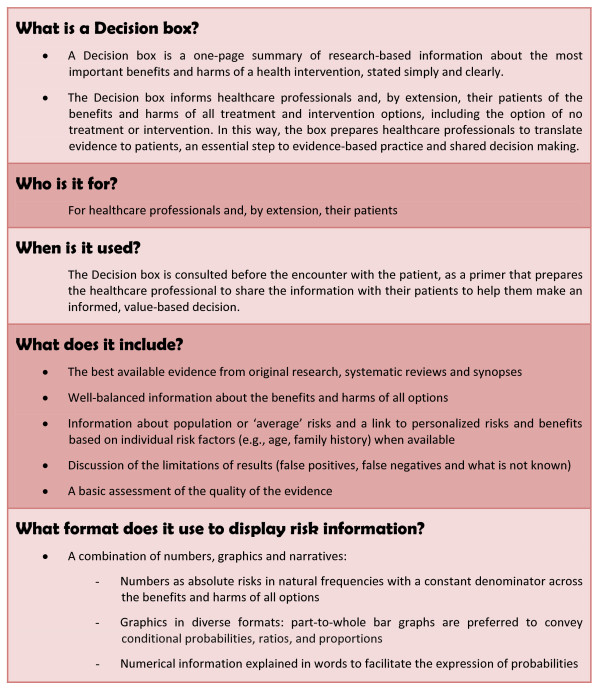
**Description of a Decision box**.

The present project aims to evaluate a novel tool, the "Decision box", which is inspired on one hand by patient decision aids in that it also aims to improve SDM in clinical practice, and on the other hand by the Drug facts box [18] in its simple and clear format to present scientific data. Short summaries of scientific information like the Drug facts box have been shown to help non-scientists better understand the benefits and side-effects of various types of medication [19]. In contrast to these two types of tools, the Decision box is primarily intended for healthcare professionals. The Decision box informs healthcare professionals, and by extension their patients, about the best available evidence on a large range of healthcare interventions (see Figure 1 for an outline). Our hypothesis is that Decision boxes will prepare clinicians to help their patients make informed, value-based decisions by giving the clinicians research-based information about the benefits and harms of various diagnostic, therapeutic, and preventive interventions, before they meet their patients. Decision boxes can be seen as primers that will enhance the application of evidence-based practice and SDM during the clinical encounter.

This paper reports on the study protocol of the first step of our research program: developing and user-testing Decision boxes. More specifically, we present (1) how we will select clinical topics for 10 Decision boxes, (2) how we will develop the Decision boxes, (3) how we will evaluate users' perception of the format and content of the Decision boxes, and (4) how we will pre-test the questionnaires to be used in the implementation study that will follow. This first study will provide a robust framework for the development and testing of Decision boxes.

## Methods

### Phase 1: Developing the Decision boxes

#### 1. Selecting the clinical topics

A panel consisting of seven of the researchers involved in this project (including four practicing family physicians) will select 10 clinical topics they perceive as relevant to primary care practice, including three relevant genomic topics. They will base their selection of the clinical topics on the following criteria:

• The health treatment or screening decision should not have a single 'best' choice, i.e., decisions should be considered 'close calls' because there is scientific uncertainty about outcomes or because a choice requires trading off benefits and harms [8].

• The clinical condition should be commonly encountered in primary care.

• A strong body of clinical research on the benefits and harms of the intervention, and ideally an appraisal of the quality of the evidence, should be available for incorporation into the Decision box.

• The intervention should be offered in at least one Canadian province.

**Figure 2:**
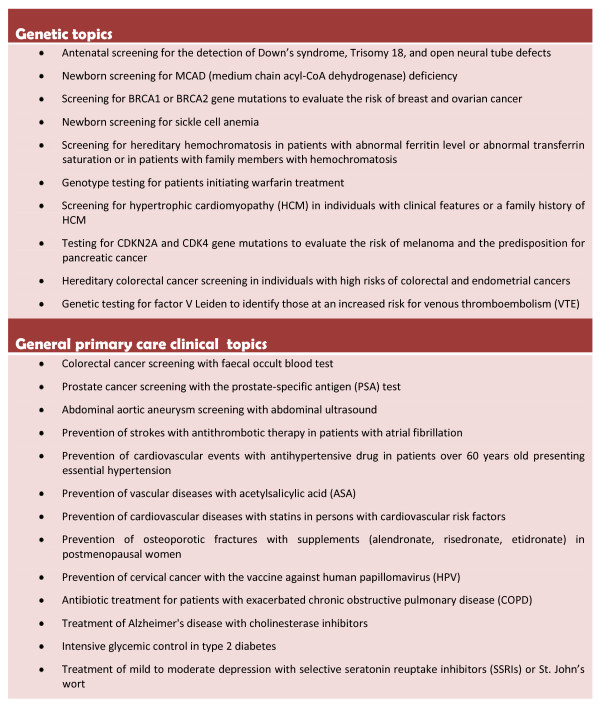
**Clinical topics to be proposed to the panelists**.

Based on a review of the literature and their clinical experience, some of the researchers in the team (ML, FR, RG) have already identified 10 genetic topics and 13 general clinical, non-genetic topics that respond to these criteria (Figure 2). We will propose these 23 topics to the panellists through a web survey. Each panellist will be asked to act independently in choosing three genetic topics and seven non-genetic topics from those proposed. Panellists will be asked to propose additional topics after the first round of the survey. At this point, we will retain the topics that were chosen by all panellists, and will remove the topics that were chosen by three panellists or less. We will then construct a new list of topics, consisting of the topics proposed by the panellists and those topics that were chosen by more than three but less than all panellists (those topics that were chosen by all panellists having already been retained). We will repeat this process until a majority of panellists have selected the same three genetic and seven non-genetic topics.

#### 2. Developing the format and content of the prototypes

**Figure 3:**
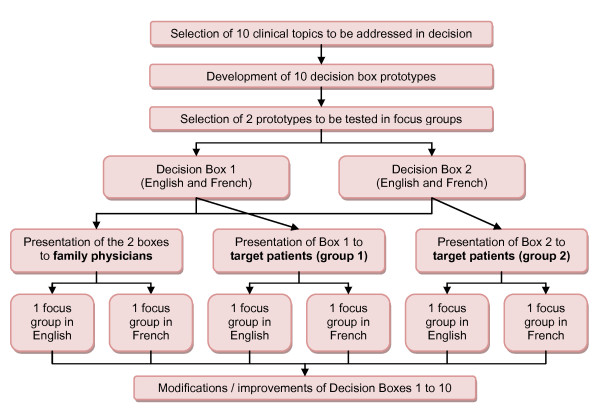
**Study procedure diagram**.

We will develop a prototype Decision box for each of the 10 topics selected. We will develop the prototypes through an iterative process that involves (1) collaboration between graphic designers and researchers, (2) field-testing by patients and clinicians, and (3) revision of the Decision box in light of the feedback provided (Figure 3). The prototypes' design will be based on the research on risk communication, as presented below. The Decision boxes will be developed in French and in English.

##### Information included in the Decision boxes

Each Decision box will begin by describing the intervention for which a decision is required. It will then identify the population targeted by the intervention and clarify the decision to be considered. The box will supply research-based information about treatment options and their benefits, harms, probabilities, and uncertainties. It will integrate the best evidence available from primary research studies, systematic reviews and synopses: for that reason, we can describe it as summary-level evidence [20,21]. The box will specify the subjects studied and the study duration. The box's design will facilitate comparison of the benefits and harms of each option and will, to the extent possible, depict the benefits and harms of each option in equal detail [22].

##### Risk communication formats

Each Decision box will present research-based information on the probabilities associated with each option as a combination of numbers, graphics and narrative statements. Although the evidence about the best risk communication formats is still not conclusive [6,23], a number of reviews recommend combining graphs with verbal and numerical formats to improve the reader's understanding of probabilistic information. Supplementing text with graphics has been proposed as a means to reduce the influence of less-relevant textual information, to improve decision-making and to increase accuracy when comparing probabilities [24]. Providing multiple formats may also help a broader audience with various numeracy levels make more accurate assessments of risks [25].

We will express the numerical probabilities of the benefits and harms of an intervention as absolute risk, communicated as natural frequencies with a constant denominator [5,6,26,27]. Natural frequencies facilitate inferences on the likelihood of having a disease because they carry implicit information about baseline risks, reduce the number of computations required to determine the positive predictive value of a test, and correspond to the way that humans have experienced statistical information for most of the history of mankind [27]. Screening test results that include false positives will be segmented into pieces (prevalence, test sensitivity, positive predictive value of a test) to improve understanding of risk estimates [28,29].

There are two reasons why graphics are recommended to present the probability of harm: graphics lead to more risk aversion than numerical probability information alone, and graphics allow the observer to process information more effectively then when numbers are presented on their own [29]. Research suggests that simple bar charts are preferable to more complex presentations of data [30]. Because no single graphical format performs optimally in all situations [31], the different Decision boxes may use different formats to display information. We will prefer part-to-whole bar graphs to convey conditional probabilities, ratios, and proportions, as these graphs are believed to invoke automatic visual area processing and proportion judgments and help viewers attend to mathematical proportions [5].

Because the Decision boxes seek to prepare clinicians to communicate probabilities to their patients, we will supplement numerical and graphical information with a short narrative statement describing the absolute risk differences between the options available [29,32].

Similarly to the Drug facts box (Dr Lisa Schwartz, personal communication), we will reserve the bottom of each Decision box to present confidence in the results, using an approach adapted from the Grading of Recommendations Assessment, Development and Evaluation (GRADE) Working Group for reporting the consistency of results, the indirectness of evidence, study limitations, and imprecision [33].

### Phase 2: User-testing the Decision boxes

#### 1. Focus groups with users

To explore users' perceptions of the content and format of the Decision boxes and to seek suggestions for improvement, we will conduct two focus groups with family physicians and four focus groups with patients (Figure 3). Physicians will be recruited by five members of the research team through their professional networks (ML, FL, MC, PP, RG). Eligible patients will be first identified by the healthcare professionals and support staff of the clinics of the network. Support staff will ask them for their permission to be contacted by a member of the research team who will then offer them to participate in the study.

To explore physicians' perceptions, we will present two Decision box prototypes to a purposeful sample of 16 family physicians distributed in two focus groups: a French-speaking group and an English-speaking group. Each focus group will comprise eight physicians. To explore patients' perceptions, we will present the same two Decision boxes to a purposeful sample of patients from each target population (the population of patients with the clinical condition addressed by the Decision box). We will use a maximum variation strategy to populate the samples [34], each of which will form a distinct focus group. Each Decision box will thus be presented to 16 patients of the target population (eight French-speaking and eight English-speaking patients, distributed in a French-speaking group and an English-speaking group), for a total of 32 patients in four focus groups.

**Figure 4:**
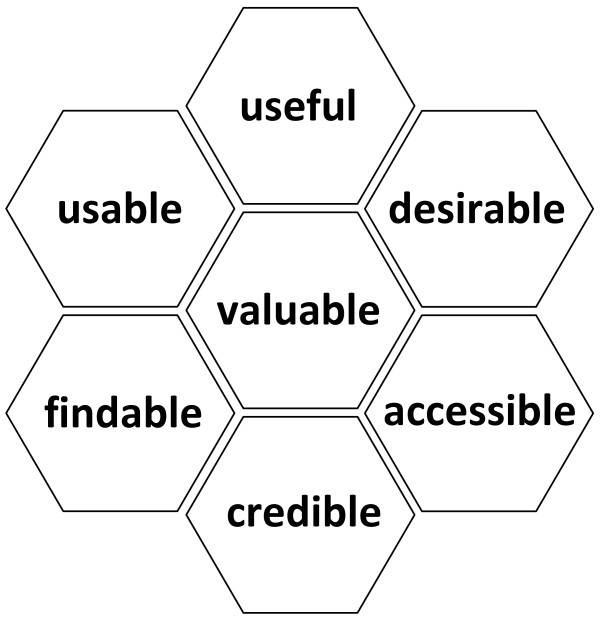
**Morville's User Experience Honeycomb **[35]. Useful: fit for practical use in the clinical setting; Usable: the extent to which target users can use the product to achieve specific goals with effectiveness, efficiency and satisfaction in a given context; Desirable: worth having and wanted by most people; Findable: the ease with which the information sought can be found within the product; Accessible: the degree to which the product can be accessed by as many people as possible; Credible: worthy of belief or trust; Valuable: able to advance the "mission" (for this study, Decision boxes are deemed valuable if they increase the use of evidence-based practice and increase shared decision making in clinical practice).

We will use a semi-structured interview guide to explore users' experience of the content and format of the Decision boxes. The interview format will be based on the User Experience Model by Peter Morville [35] as used for testing the Cochrane Collaboration's Summaries of Findings table [36]. This model explores seven facets of users' experience (Figure 4). The interview guide will be flexible and will cover all key topics for all focus groups, although not necessarily in the same order (interviews will follow the natural progression of the conversation). The interview format will give participants the flexibility to explore emerging issues. The interview guide used for patients will differ slightly from that for physicians. The main objective of interviewing the physicians is to explore the value of the tool in preparing them to communicate scientific information to patients and helping patients make informed and value-based decisions. The objective of the interview with patients will be to explore whether patients believe that their physician will be better prepared to meet them having read the box beforehand, and to ask them whether the box contains all the information they need to make a decision. After the focus group with patients, they will be administered the Decisional Conflict Scale [37], and patients with a decisional conflict measure above 2.5 over 5 will be advised to see their doctor for follow-up.

To enrich our understanding of the group's views, we will collect demographic data from all participants at the beginning of each focus group. The focus groups with physicians will last approximately two hours; those with patients will last approximately one hour. The focus groups will be moderated by experienced interviewers (one for the French-speaking groups and one for the English-speaking groups). Two observers (AG, PP or ML) will take notes on the process and the content of the discussions so that participants can be identified when the interviews are transcribed [38]. One observer (AG) will be present during every focus group discussion, to ensure consistency of approach. Each discussion will be audiotaped and professionally transcribed. Ethical approvals for this project were given by the research ethics committees of the Centre de Recherche du Centre Hospitalier Universitaire de Quebec (reference number #S10-12-114) and by McGill University (reference number #A12-E82-10B).

#### 2. Testing the feasibility of the questionnaire for family physicians

Following their focus group, family physicians will be asked to pre-test a self-administered questionnaire to be used in a larger study on the implementation of the Decision boxes, to take place later. Pretesting will allow us to evaluate physicians' understanding of the content of the questionnaire and the feasibility of having the physicians complete the questionnaire under field conditions. The questionnaire will be made available in English and in French. It will collect the respondent's sociodemographic characteristics and will evaluate the respondent's interest in the clinical topic using a visual analogue scale ranging from "no interest" to "deep interest". The questionnaire will include the information sub-scale of the Decisional Conflict Scale [37], the Information Assessment Method [39], and a scale based on the Theory of Planned Behaviour to evaluate physicians' intention to use the Decision boxes in their practice and to share decisions equally with their patients [40]. Open-ended questions on the relevance of the questions and respondents' ease of understanding will be added at the end. We will record the time needed for physicians to complete the questionnaire.

#### 3. Analysis

Two individuals (one research assistant and one researcher) will independently perform a hybrid deductive/inductive thematic qualitative data analysis of each focus group discussion using specialized software (NVivo 9) [41]. A first analysis will be performed by a research assistant (one in French, one in English), and a second analysis will be performed by one of the researcher (AG). Any disagreements between the research assistants and the researcher will be discussed until consensus is reached. Having the same researcher perform the second analysis for all of the sites will ensure consistency. The coding scheme will be developed following the User Experience Model mentioned earlier [35] by identifying what users experienced as barriers or facilitators to the use of the Decision boxes and using this information to help clinicians share decisions with their patients. The deductive thematic analysis will apply attributes derived a priori from the seven facets of the User Experience Model [35]: valuable, usable, credible, useful, desirable, accessible and findable. The inductive thematic analysis will integrate new themes, suggested by the data, into the scheme. We will compare the phenomena observed to emphasize a common tangent and will work out tree structures and matrices for the analysis. Other members of the research team will corroborate the findings by scrutinizing the analysis and ensuring that the new themes, tree structures and matrices are representative of the initial data analysis and codes assigned. We will triangulate key findings by referring to publications on the design of tools to improve the communication of risk [23,42].

We will also perform descriptive statistical analyses of the answers to the questionnaire. To test the feasibility of the questionnaire, we will evaluate response rates and the time required to complete the questionnaire. We will also perform a descriptive analysis of respondents' comments about the relevance of the questions and how well they understood them.

## Discussion

This study will create a framework for the production of Decision boxes designed to facilitate decision making about preference-sensitive health interventions. It will produce 10 bilingual Decision boxes on common clinical topics in primary care, and it will generate robust evidence of family physicians' and patients' perceptions of the content and format of Decision box prototypes. This preliminary work is crucial to the implementation of Decision boxes in clinical practice.

The methodology proposed here has several strengths. First, we will ground our development of the Decision boxes in a thorough review of the rich evidence base on risk communication. Second, we will develop the boxes in both of Canada's official languages and have them tested by English-speakers and French-speakers alike. And third, we will have the boxes tested by both parties to SDM during the clinical encounter: that is, by both family physicians and patients. The limitations of our study lie in users' evaluation of only two of the 10 Decision boxes we will have developed, a limitation necessitated by the restricted availability of funds. The authors are confident, however, that many of the comments gathered on the two Decision boxes-namely, comments regarding the boxes' format-will apply to Decision boxes on any clinical topic.

We plan to conduct another study that evaluates the effect of the Decision boxes on the integration of evidence-based and SDM principles in practice. We hypothesize that exposing family physicians to a theory-based strategy [46] to implement Decision boxes will prime the physicians to better communicate the benefits and harms of the available options to their patients. This communication will cause patients to become more involved in decisions concerning their health. By helping to implement Decision boxes in clinical practice, the subsequent steps of this research program will impact not only the communication of scientific data to physicians, but also the communication between physicians and patients, leading physicians to make more judicious use of current best evidence when making clinical decisions together with their patients.

## Competing interests

The authors declare that they have no competing interests.

## Authors' contributions

All authors contributed to the study plan. AG wrote the first draft of the manuscript, and AG and ML reviewed it at various stages to its final version. All authors reviewed the manuscript and approved its final version.

## Pre-publication history

The pre-publication history for this paper can be accessed here:

http://www.biomedcentral.com/1472-6947/11/17/prepub
